# 51例伴11q异常的高级别B细胞淋巴瘤患者临床病理特征及疗效的回顾性分析

**DOI:** 10.3760/cma.j.cn121090-20260310-00127

**Published:** 2026-06

**Authors:** 璐 王, 歌 高, 依萍 靳, 道远 吴, 璐阳 常, 旭东 魏, 可树 周, 庆欣 夏

**Affiliations:** 1 郑州大学附属肿瘤医院，河南省肿瘤医院病理科，郑州 450008 Department of Pathology/Henan Cancer Hospital, Affiliated Cancer Hospital of Zhengzhou University, Zhengzhou 450008, China; 2 河南省肿瘤病理和人工智能诊断医学重点实验室，郑州 450008 Henan Key Laboratory of Tumor Pathology and AI-based Diagnostic Medicine, Zhengzhou 450008, China; 3 河南省病理诊断抗体工程研究中心，郑州 450008 Henan Engineering Research Center of Antibody for Pathological Diagnosis, Zhengzhou 450008, China; 4 郑州大学附属肿瘤医院，河南省肿瘤医院血液科，郑州 450008 Department of Hematology/Henan Cancer Hospital, Affiliated Cancer Hospital of Zhengzhou University, Zhengzhou 450008, China

## Abstract

为探讨伴11号染色体长臂（11q）异常的高级别B细胞淋巴瘤（HGBCL-11q）的临床病理特征、分子遗传学特点及不同强度化疗方案的疗效，本研究回顾性分析就诊于河南省肿瘤医院的51例HGBCL-11q患者资料，总结其临床及病理分子特征，比较不同治疗强度组间的疗效与生存差异。结果显示，51例患者中位年龄52（5～84）岁，男女比例为1.22∶1，消化系统为最常见发病部位（18/51，35.3％）。肿瘤细胞呈生发中心B细胞免疫表型，47例（92.2％）肿瘤细胞不同程度表达c-Myc（阳性细胞占比20％～85％），41例（80.4％）不表达Bcl-2，47例（92.2％）Ki-67增殖指数≥90％。荧光原位杂交检测提示无MYC基因重排，特征性出现11q异常，高频突变基因包括DDX3X（60.0％，12/20）、PCLO（50.0％，10/20）、TP53（45.0％，9/20）、GNA13（40.0％，8/20）等。含利妥昔单抗的高强度治疗组完全缓解率显著高于标准治疗组［87.5％（21/24）对60.9％（14/23），*P*＝0.036］，且无进展生存期更长［（62.7±3.9）个月对（41.7±6.5）个月，*P*＝0.014］。HGBCL-11q具有特征性临床病理表现及分子遗传学特征，含利妥昔单抗的高强度化疗可显著提高缓解率并改善生存。

## Abstract (en)

To investigate the clinicopathological features, molecular genetic characteristics, and efficacy of chemotherapy regimens with different intensities in high-grade B-cell lymphoma with aberrations in the long arm of chromosome 11（HGBCL-11q）, this study retrospectively analyzed the data of 51 patients with HGBCL-11q at Henan Cancer Hospital, summarized their clinical, pathological and molecular profiles, and compared the differences in therapeutic efficacy and survival outcomes between groups with different treatment intensities. The median age of the 51 patients was 52（5–84）years, with a male-to-female ratio of 1.22:1, and the digestive system was the most common site of involvement（18/51, 35.3％）. The tumor cells exhibited a germinal center B-cell immunophenotype: 47 patients（92.2％）showed variable c-Myc expression in tumor cells（the proportion of positive cells ranged from 20％ to 85％）, 41 patients（80.4％）were negative for Bcl-2 expression, and 47 patients（92.2％）had a Ki-67 proliferation index ≥90％. Fluorescence in situ hybridization confirmed the absence of MYC gene rearrangement and the presence of characteristic aberrations in 11q; recurrently mutated genes included DDX3X（60.0％, 12/20）, PCLO（50.0％, 10/20）, TP53（45.0％, 9/20）and GNA13（40.0％, 8/20）, et al. The complete response rate in the rituximab-containing high-intensity chemotherapy group was significantly higher than that in the non-high-intensity group［87.5％（21/24）*vs* 60.9％（14/23）, *P*＝0.036］, with longer progression-free survival［（62.7±3.9）months *vs*（41.7±6.5）months, *P*＝0.014］. In conclusion, HGBCL-11q has distinctive clinicopathological manifestations and molecular genetic features, and rituximab-containing high-intensity chemotherapy can significantly improve the response rate and survival of patients.

伴11号染色体长臂（11q）异常的高级别B细胞淋巴瘤（HGBCL-11q）于2022年第五版世界卫生组织（WHO）分类中被正式确立为一种独立的高级别成熟B细胞肿瘤实体，其分子遗传学特征为11q23.2-23.3区域扩增和（或）11q24.1-qter区域缺失，不伴有MYC、Bcl-2、Bcl-6基因断裂[1]–[2]。该病发病率低，流行病学特征、最佳治疗策略及预后仍不明确。有研究报道HGBCL-11q可发生于各年龄段，以儿童和青年多见，可能具有独特的免疫表型特征（如LMO2阳性、CD38阴性）和相对较好的预后趋势[3]–[5]。本研究汇总51例HGBCL-11q患者的临床资料，分析其发病部位、临床分期、分子病理特点等，为该疾病的诊疗与预后评估提供临床依据。

## 病例与方法

1. 病例：本研究是一项回顾性队列研究，纳入2017年1月至2025年12月于河南省肿瘤医院确诊的51例HGBCL-11q患者的临床资料。所有病例均经组织病理学、免疫组织化学染色及荧光原位杂交（FISH）检测确诊，符合WHO淋巴造血系统肿瘤分类的诊断要求并经过2位淋巴瘤专业组诊断医师复验。HIV相关淋巴瘤与既往接受过系统性抗淋巴瘤治疗者被排除。收集患者人口学特征、临床特征、实验室指标、病理与分子特征、治疗及生存预后等资料，按国际预后指数（IPI）标准进行风险分层（IPI评分0～1分为低危、2分为低中危、3分为中高危、4～5分为高危）。本研究经河南省肿瘤医院医学伦理委员会批准（批件号：2026-336-001），并豁免患者知情同意。

2. 免疫组织化学染色：肿瘤组织经3.7％中性甲醛固定，全自动脱水机脱水处理，石蜡包埋，4 µm厚切片，予以苏木精-伊红染色法（HE）染色，光镜分析。免疫组化采用EnVision法，经高压热修复抗原、3％过氧化氢阻断内源性过氧化物酶，依次滴加一抗（4 °C孵育过夜）及相应二抗，DAB显色后苏木素浅染复核。所用一抗均为福州迈新生物技术开发有限公司即用型试剂，无需稀释，抗体及对应货号为CD20（Kit-0001）、CD3（MAB-0740）、CD10（MAB-0668）、Bcl-2（RMA-0660）、Bcl-6（MAB-0746）、c-Myc（RMA-0803）、TDT（MAB-0676）、Ki-67（RMA-0542）。

3. FISH检测：切片经预处理、胃蛋白酶消化、高温变性后与荧光标志探针于37 °C杂交过夜，经严格洗涤后以4′,6-二脒基-2-苯基吲哚（DAPI）复染细胞核，荧光显微镜下判读结果。FISH检测所用探针均为广州安必平医药科技股份有限公司生产，方法参考文献[6]，步骤详见产品说明书。其中，11q23.3/11q24.3基因缺失检测使用相应探针（No. F.01391-01），11q23.3位点标记为红色，11q24.3位点标记为绿色。正常细胞显示2红2绿信号；若超过20％的细胞出现3红1绿或2红1绿的信号模式，则判定为11q异常阳性。使用MYC（No. F.01006-01）、Bcl-2（No. F.01172-01）及Bcl-6（No. F.01069-01）分离探针检测基因断裂情况，若观察到红绿分离信号，提示相应基因发生断裂重组。

4. 高通量测序靶向检测基因突变：我院病理科采用赛默飞世尔科技（中国）有限公司的S5系统对提取的肿瘤组织DNA进行基因突变分析，使用我院针对淋巴瘤相关基因设计的探针panel，建库后于Illumina测序平台上进行高通量测序，平均测序深度≥500×。原始数据经质控后，与人类参考基因组（hg38）进行比对，利用基因组分析工具包（GATK）等标准流程进行单核苷酸变异（SNV）、插入缺失（Indel）等变异的识别。所有检出的变异均依据文献[7]及公共数据库进行致病性注释与临床意义解读。

5. 治疗方案和疗效评估：所有患者均接受利妥昔单抗联合化疗作为初始诱导治疗，治疗方案包括利妥昔单抗联合高强度化疗［hyperCVAD方案（环磷酰胺+长春新碱+多柔比星+地塞米松，交替甲氨蝶呤+阿糖胞苷）、R-IVAC方案（利妥昔单抗+异环磷酰胺+依托泊苷+阿糖胞苷）、R-EPOCH方案（利妥昔单抗+依托泊苷+泼尼松+长春新碱+环磷酰胺+多柔比星）、R-NHL-BFM方案（利妥昔单抗联合BFM分层化疗）等］、标准方案［R-CHOP方案（利妥昔单抗+环磷酰胺+多柔比星+长春新碱+泼尼松）、R-CHP（利妥昔单抗+环磷酰胺+多柔比星+泼尼松）+维泊妥珠单抗等］。对于诱导治疗获得完全缓解（CR）或部分缓解（PR）的高危、进展期患者，根据患者年龄及意愿选择序贯auto-HSCT或完成6～8个疗程诱导治疗。根据2014年Lugano疗效评价标准对患者进行疗效评估，疗效评估节点包括治疗中期（2～4个疗程后）、治疗结束后和随访期。治疗效果分为CR、PR、疾病稳定（SD）和疾病进展（PD），客观缓解率（ORR）为CR率和PR率之和。

6. 随访：通过查阅病历和电话进行随访，截止日期为2025年12月31日。总生存（OS）期定义为自明确诊断至任何原因导致死亡或末次随访时间，无进展生存（PFS）期定义为自接受治疗至首次出现疾病进展、或因任何原因死亡或末次随访时间。

7. 统计学处理：应用SPSS 26.0统计学软件进行数据处理。符合正态分布的计量资料用*x*±*s*描述，非正态分布的计量资料用*M*（范围）表示，计数资料用例（％）表示，组间数据的比较采用卡方检验或Fisher确切概率检验。Kaplan-Meier方法进行生存分析。*P*<0.05为差异有统计学意义。

## 结果

1. 患者一般临床特征：如表1所示，研究共纳入51例HGBCL-11q患者，中位年龄52（5～84）岁，男女比例1.22：1。患者原发受累部位以消化道最为多见（35.3％，18/51），其次为浅表淋巴结（23.5％，12/51），仅少数患者合并骨髓或中枢神经系统受累（19.6％，10/51）；Ann Arbor分期Ⅰ～Ⅱ期27例（52.9％），IPI评分以低危和低中危为主（68.6％，35/51）；B症状发生率较低（11.8％，6/51），超过半数患者血清乳酸脱氢酶>220 U/L（60.8％，31/51）及β_2_微球蛋白≥2.0 mg/L（52.9％，27/51）。

**表1:** 51例伴11q异常的高级别B细胞淋巴瘤患者临床特征

临床特征	例（％）
性别	
男性	28（54.9）
女性	23（45.1）
年龄（岁）	
≤18	9（17.7）
19～59	27（52.9）
≥60	15（29.4）
Ann Arbor分期	
Ⅰ～Ⅱ期	27（52.9）
Ⅲ～Ⅳ期	24（47.1）
IPI评分分层	
低危（0～1分）	26（51.0）
低中危（2分）	9（17.6）
中高危（3分）	8（15.7）
高危（4～5分）	8（15.7）
B症状	
无	45（88.2）
有	6（11.8）
骨髓或中枢神经系统受累	
无	41（80.4）
有	10（19.6）
ECOG评分	
0分	17（33.3）
1分	31（60.8）
≥2分	3（5.9）
发病部位	
消化道	18（35.3）
浅表淋巴结	12（23.5）
深部淋巴结	5（9.8）
头颈部	6（11.8）
中枢神经系统	1（2.0）
其他	4（7.8）
混合	5（9.8）
LDH	
>220 U/L	31（60.8）
≤220 U/L	20（39.2）
β_2_微球蛋白	
<2.0 mg/L	23（45.1）
≥2.0 mg/L	27（52.9）
未知	1（2.0）

**注** IPI：国际预后指数；ECOG：美国东部肿瘤协作组；LDH：乳酸脱氢酶

2. 患者病理特征：患者肿瘤细胞中等大小，相对一致，"星空现象”易见（图1A），47例（92.2％）患者Ki-67增殖指数≥90％。所有患者肿瘤细胞均表达CD20，不表达CD3、CD30，而生发中心标志物CD10和Bcl-6表达率分别为72.5％（37/51）、90.2％（46/51）。47例（92.2％）患者肿瘤细胞表达c-Myc（阳性细胞数占比区间为20％～85％），41例（80.4％）患者不表达Bcl-2。FISH检测结果证实51例患者均存在典型的11q扩增或缺失（图1B），MYC、Bcl-2和Bcl-6基因均未检测到异常断裂信号。

**图1:**
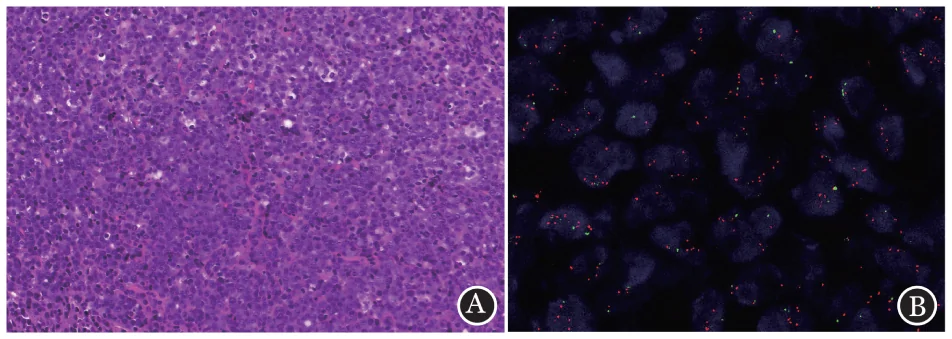
伴11q异常的高级别B细胞淋巴瘤（HGBCL-11q）的组织病理学特征及FISH检测结果 **A** HGBCL-11q淋巴结结构破坏，肿瘤细胞弥漫生长，“星空现象”易见（HE染色，×400）；**B** 11q23.3/11q24.3基因缺失探针（荧光原位杂交）结果显示11q23.3位点（红色信号）发生扩增，同时11q24.3位点（绿色信号）发生缺失（×1 000）

研究共收集到20例患者的测序结果，检出率≥15.0％（3/20）的基因突变共18种，突变检出率排名前五的依次为DDX3X（60.0％，12/20）、PCLO（50.0％，10/20）、TP53（45.0％，9/20）、GNA13（40.0％，8/20）、MLL2（25.0％，5/20）。部分基因存在共现现象，DDX3X与TP53、DD3X3与PCLO共突变6例，TP53与PCLO共突变5例，而4例患者同时存在DDX3X、TP53、PCLO基因突变（图2）。

**图2:**
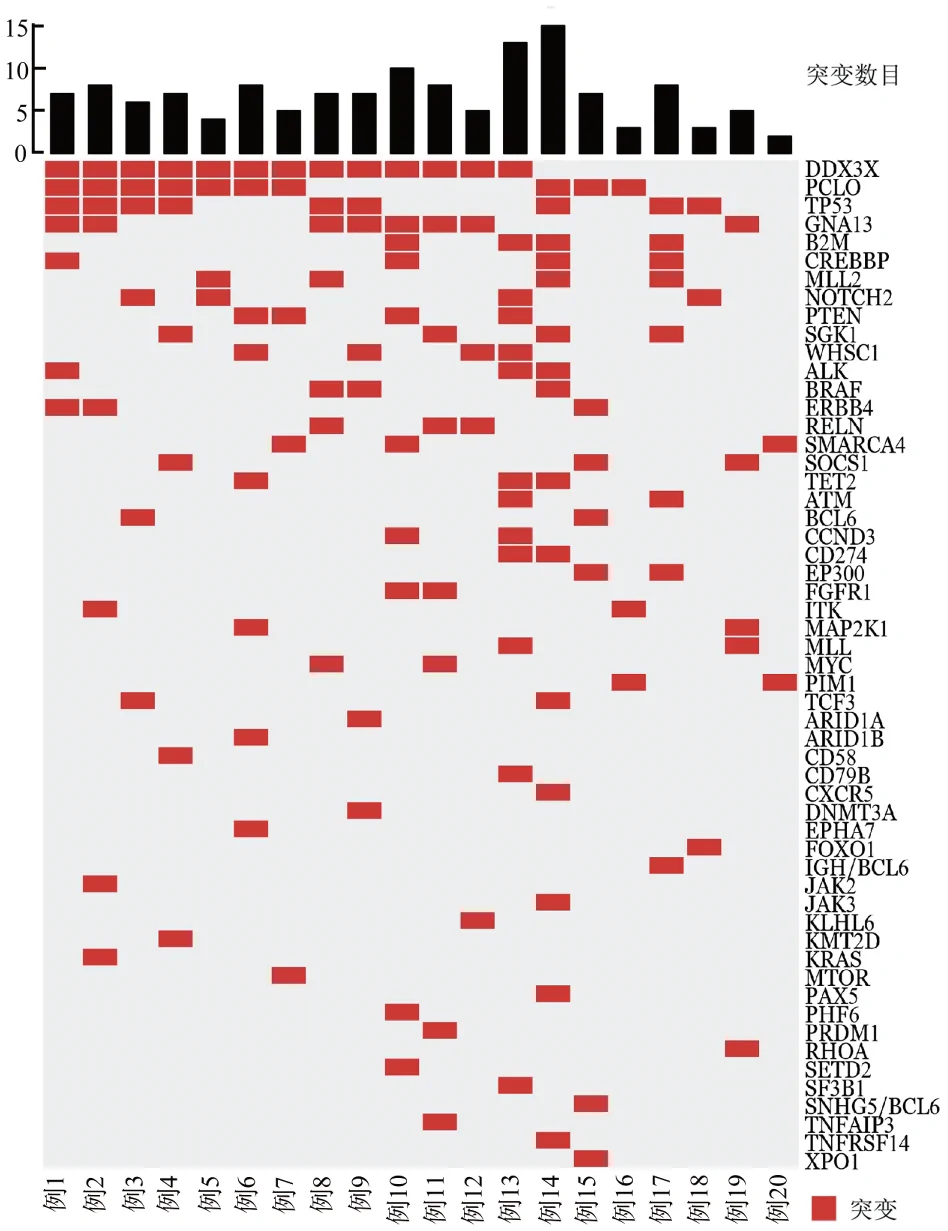
20例伴11q异常的高级别B细胞淋巴瘤患者基因突变分布图

3. 治疗方案与疗效：51例患者均接受含利妥昔单抗的治疗。27例（52.9％）接受标准方案治疗（标准治疗组），24例（47.1％）接受高强度化疗（高强度治疗组），两组患者IPI危险度分层构成差异无统计学意义（*P*>0.05）。4例患者未完成疗效评估，余47例患者ORR为80.9％（38/47），CR率为74.5％（35/47）。高强度治疗组的CR率高于标准治疗组［87.5％（21/24）对60.9％（14/23），*χ*²＝4.381，*P*＝0.036］。9例青少年患者均接受高强度化疗且达CR，除1例在2年后中枢神经系统复发，其余患者均在末次随访时保持CR状态。

47位患者中位随访时间为26（6～71）个月，总体OS期为（64.6±3.0）个月，高强度治疗组患者OS期长于标准治疗组，但差异无统计学意义［（68.5±2.4）个月对（54.9±4.9）个月，*P*>0.05］。患者总体PFS期为（54.1±4.2）个月，高强度治疗组患者PFS期较标准治疗组更长［（62.7±3.9）个月对（41.7±6.5）个月，*P*＝0.014］。

## 讨论

本研究纳入的51例经FISH证实的HGBCL-11q患者中位年龄为52岁，其中≤18岁者仅占17.6％，这一发现与日本Yamada等[3]报道的年龄分布相似，但与谢建兰等[4]以儿童青少年为主的结论有所不同，提示HGBCL-11q的发病年龄谱可能比既往认知更广泛。消化系统为本病最常见起病部位，其次为浅表淋巴结，与谢建兰等[4]报道的19例中国患者结外病变高发的特点一致。临床分期上，Ⅰ～Ⅱ期与Ⅲ～Ⅳ期患者比例接近，IPI评分低危患者占半数，提示部分患者就诊时疾病处于早期阶段，为临床早期干预提供了有利条件，可能是其整体预后相对良好的基础。

病理学方面，患者总体肿瘤细胞均表达CD20，不表达CD3、CD30，CD10和Bcl-6阳性率分别为72.5％和90.2％，符合生发中心B细胞（GCB）来源表型。c-Myc蛋白表达率达92.2％，但阳性强弱程度不一，FISH检测未见MYC基因重排，这可能是其形态类似伯基特淋巴瘤却缺乏典型遗传学改变的重要原因；c-Myc蛋白高表达而Bcl-2低表达是区别于双重打击淋巴瘤的重要免疫表型线索[8]–[9]。

既往研究证实DDX3X与GNA13为HGBCL-11q的高频突变基因，是核心驱动事件；同时HGBCL-11q缺乏经典伯基特淋巴瘤特征性的ID3/TCF3通路突变，遗传学特征更接近GCB型弥漫大B细胞淋巴瘤[9]–[11]。本研究结果与此一致，验证了上述分子标志物在HGBCL-11q中的普遍性。本研究还发现PCLO、TP53及MLL2亦为HGBCL-11q的高频突变基因，且DDX3X、TP53与PCLO三者存在频繁共突变现象，丰富了对其基因突变谱的认识。生物学机制方面，DDX3X编码的RNA解旋酶参与mRNA代谢与细胞周期调控，GNA13则是生发中心B细胞存活与迁出的关键调节因子，二者的失活突变是HGBCL-11q重要的致病基础[12]–[15]。本研究中TP53突变检出率较高，其功能缺失突变与淋巴瘤化疗耐药、不良预后直接相关，且TP53与DDX3X共突变可界定弥漫大B细胞淋巴瘤中预后极差的高侵袭性亚群，二者的协同作用进一步加剧疾病进展与治疗抵抗[16]。结合PCLO突变与B细胞淋巴瘤化疗耐药相关的既往报道，推测DDX3X、TP53与PCLO的协同失活可能构成了HGBCL-11q特有的致病通路，或可作为未来疾病风险分层的潜在分子标志物[17]–[18]。

HGBCL-11q仍缺乏大规模前瞻性临床研究数据支持，其治疗策略尚未形成统一共识，现有治疗经验主要来源于小规模回顾性研究及个案报道。本研究结果显示，与接受标准强度方案患者相比，接受含利妥昔单抗的高强度化疗的患者具有更高的CR率与更长的PFS期，且青少年患者对高强度方案耐受性良好。文献回顾亦支持高强度化疗在HGBL-11q中的优势地位，如2例青少年HGBL-11q患者，采用利妥昔单抗联合HyperCVAD方案治疗后均获CR，且随访期间无复发[19]。1例原发于心脏的HGBL-11q患者初期采用SMART方案（利妥昔单抗联合来那度胺、奥布替尼）疗效不佳，更换为R-Hyper-CVAD方案后获得PR，提示靶向药物联合方案可能不足以控制高侵袭性病变，强化疗仍是核心手段[20]。本研究中仍有近半数患者接受了以R-CHOP方案为代表的标准强度治疗，反映该类罕见疾病临床认知存在局限性。既往多数形态学符合高级别B细胞淋巴瘤但MYC等重排阴性的病例常被归入弥漫大B细胞淋巴瘤，对其侵袭性生物学行为认识不足，进而导致治疗强度不足、复发风险升高[1],[8]–[9]。本研究结果提示需重视11q异常的常规筛查，对明确诊断的HGBCL-11q患者优先选择高强度化疗方案；同时结合TP53等高危突变的检测结果，进一步为患者制定个体化的治疗策略。

本研究为回顾性分析，样本量较小且基因突变数据仅覆盖部分病例，未来需开展多中心前瞻性研究验证高强度化疗的疗效，探索DDX3X、TP53、GNA13等基因突变对治疗反应及预后的影响。综上，HGBCL-11q呈现年龄范围谱广泛、结外病变高发于消化系统的临床特征，具有典型的GCB表型、特征性的11q异常和以DDX3X、TP53、GNA13等为核心的高频突变特征性基因突变谱；早期应用含利妥昔单抗的高强度化疗可提高患者CR率和延长PFS期。
